# Disproportionate utilization of healthcare resources among veterans with COPD: a retrospective analysis of factors associated with COPD healthcare cost

**DOI:** 10.1186/1478-7547-11-13

**Published:** 2013-06-13

**Authors:** Kyle Darnell, Alok Kumar Dwivedi, Zhouyang Weng, Ralph J Panos

**Affiliations:** 1 Pulmonary, Critical Care, and Sleep Medicine Division, Cincinnati Veterans Affairs Medical Center, Cincinnati, OH 45220, USA; 2 Pulmonary, Critical Care, and Sleep Medicine Division, University of Cincinnati College of Medicine, Cincinnati, OH 45267, USA; 3 Division of Biostatistics and Epidemiology, University of Cincinnati College of Medicine, Cincinnati, OH 45267, USA

**Keywords:** COPD, Veterans healthcare administration, Healthcare utilization, Cost

## Abstract

**Background:**

COPD is a significant cause of morbidity and mortality in the Veterans Health Administration (VHA). To determine the clinical factors associated with the cost of COPD management, we analyzed the relationship between clinical characteristics and COPD healthcare costs at the Cincinnati VAMC.

**Methods:**

We queried the VHA Decision Support System for patients diagnosed with COPD at the Cincinnati VAMC and calculated their VHA COPD-related encounters and costs in FY2008. Patients were ranked by COPD-related cost. We determined the detailed clinical characteristics of patients selected by modified systematic sampling and performed univariate and multivariable ordinary linear regression analysis to determine factors associated with cost.

**Results:**

3263 Veterans had 11,869 encounters with a primary or secondary diagnosis of COPD: 10,032 clinic visits, 505 emergency department (ED) visits, and 1,332 hospitalizations and incurred a total COPD-related healthcare cost of $21.4 M: $2.4 M clinic visits, $0.21 M ED visits, and $18.7 M hospitalizations and $0.89 M for COPD-related prescription costs. When the patients were ranked by VHA healthcare costs, the top 20% of patients accounted for 86% of the total costs and 57% of the total encounters with a primary or secondary diagnosis code of COPD and 90% of the total costs and 75% of the total encounters with a primary diagnosis code of COPD. The clinical characteristics and VHA healthcare costs of 840 of the 3263 unique individuals with COPD were analyzed to determine those characteristics associated with increased COPD-related costs. Univariate analysis showed significant associations with 24 clinical variables; the 4 most highly associated factors were nursing home residence, total hospital admissions, use of oral corticosteroids, and supplemental oxygen (p < 0.001 for all). In multivariate analysis, total number of admissions (p < 0.001), management by a pulmonologist (p < 0.001), number of clinic visits (p < 0.001), use of short acting anticholinergic (p = 0.001), forced expiratory volume in 1 second (FEV1) (p = 0.011), number of prescriptions (p = 0.011), body mass index (BMI) (p = 0.025), and use of inhaled corticosteroid (p = 0.043) were associated with COPD management cost.

**Conclusion:**

The total number of admissions, clinic visits, physiologic impairment, BMI, number of medications, and type of provider are strongly associated with the total cost of COPD management. These factors may be used to focus COPD management toward patients with the potential for high utilization of healthcare resources.

## Introduction

Chronic obstructive pulmonary disease (COPD) is a progressive, debilitating lung disorder characterized by non-normalizing airflow limitation. At the Cincinnati Veteran’s Administration Medical Center (VAMC), the prevalence of airflow limitation is estimated to be 33-43% and COPD is significantly under-diagnosed and misdiagnosed [1]. In a 1996–2001 utilization review, 19% of men and 17% of women who received care from the Veterans Healthcare Administration (VHA) were diagnosed with COPD and COPD was the fourth most common discharge diagnosis at VHA hospitals [2]. Further, COPD care is a major expense; the VHA spent an estimated $5.5 billion to care for approximately 969,000 Veterans with COPD in 2004 [3]. COPD is a common disorder of Veterans that causes significant morbidity and mortality and its treatment is a major expense within the VHA.

The clinical course of COPD is marked by acute exacerbations (AECOPD) that often precipitate healthcare encounters. After an AECOPD, patients frequently do not recover to the prior level of lung function, and have a lower quality of life and reduced survival compared with individuals who do not have AECOPD’s [4-6]. AECOPD’s account for approximately 40% of all direct Medicare expenses for the treatment of COPD [5,7] and the number of hospitalizations is a primary contributor to COPD-related healthcare costs [8,9]. Numerous clinical variables have been associated with hospital admissions for AECOPD [7,10-25]. Reduced pulmonary function, prior hospitalizations for AECOPD, and use of systemic steroids are the most frequently identified predictors of hospitalization for AECOPD.

The distribution of healthcare costs among Veterans with COPD and the clinical characteristics that are associated with greater costs have not been well studied within the VHA [26,27]. The goal of this study was to determine the clinical characteristics of Veterans with COPD that associate with COPD-related and total VHA healthcare costs and their utilization of VHA resources for COPD management.

## Methods

### Study design and patient population

We queried the VHA Decision Support System (DSS) for the names of all Veterans with a primary or secondary diagnosis of COPD (496.xx), chronic bronchitis (491.xx), and emphysema (492.xx) who received care at the Cincinnati VAMC in the fiscal year 2008 (October 1, 2007 to September 31, 2008) and calculated their individual VHA total and COPD-related healthcare encounters and costs. Healthcare encounters were defined by DSS variables: outpatient visits, ED visits not associated with hospitalization, and hospitalizations. We defined encounters and cost for which the primary or secondary diagnosis was COPD (496.xx), chronic bronchitis (491.xx), or emphysema (492.xx) as COPD-related. These patients were ranked by COPD-related health costs and grouped by quintiles.

**Figure 1:**
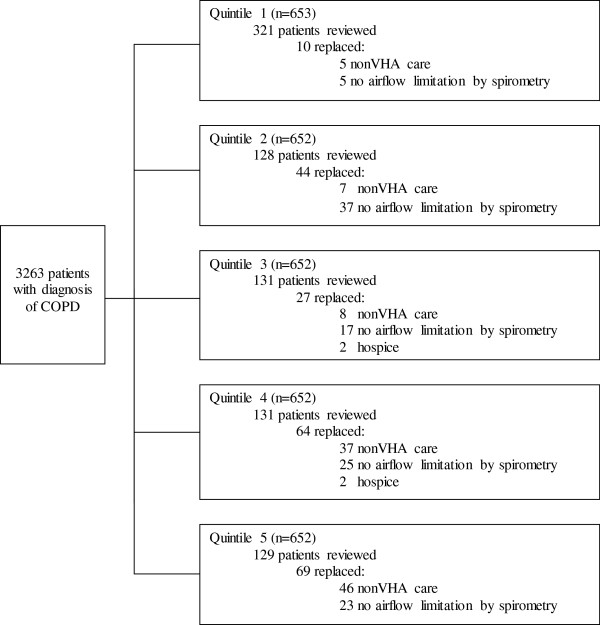
**Patient selection diagram.** Patients were selected by a modified stratified systematic sampling: every other patient in the first quintile with the greatest COPD-related healthcare costs and every fourth patient in the remaining quintiles. If a patient received primary or pulmonary care from non-VA providers, airflow limitation (FEV_1_/FVC < 0.7) was not present on spirometry, or if they were in hospice care, they were excluded and replaced by the next patient on the rank list.

After reviewing the literature, we identified clinical factors associated with healthcare costs related to COPD (Additional file 1). Patients were selected by a modified stratified systematic sampling: every other patient in the quintile with the greatest COPD-related healthcare costs and every fourth patient in the remaining quintiles. If a patient received primary or pulmonary care from non-VA providers, airflow limitation (FEV_1_/FVC < 0.7) was not present on spirometry, or if they were in hospice care, they were excluded and replaced by the next patient on the rank list (Figure 1). We reviewed each patient’s complete VHA electronic medical record including progress notes (inpatient, outpatient and nursing home), discharge summaries, medication lists, pulmonary function tests, echocardiographic data, and phone notes for demographic data and 16 risk factors for COPD exacerbations.

This study was approved by the University of Cincinnati Institutional Review Board and the Cincinnati VAMC Research and Development Committee.

### Statistical analysis

All quantitative variables are described using appropriate summary statistics (mean, median, standard deviation (SD), interquartile range (IQR) and range); categorical variables are presented using frequency and proportions. COPD-related cost and total health care cost were the primary outcomes. Initial healthcare encounter and cost analysis were performed using the DSS data from the entire population and the remaining analysis used the sampled subpopulation. Both primary outcomes were log transformed to normalize their distributions. Univariate associations of cofactors with log transformed health care costs were assessed using ordinary linear regression. Multicollinearity was determined by calculating variance inflation factors (VIF). Variables with VIF greater than 10 were considered to be multicollinear factors. However, no variable was identified as a multicollinear variable. Stepwise multiple linear regression was used to identify the adjusted association of cofactors with the outcomes. The cofactors identified in the univariate regression analyses at 10% level of significance were included in the stepwise multivariable linear regression analysis. The inclusion and exclusion criteria were set at 5% and 10%, respectively, in the stepwise regression. The multiple linear regression results are shown using adjusted regression coefficients and their 95% confidence intervals (CI) and p-values. The multiple linear regression analyses were again developed after excluding the probable outliers. However, there was no change observed in the results with or without outliers. Thus, the final multiple linear regression analysis was conducted on all the available data points. The multiple linear regression analyses were also conducted after excluding the variables that had more missing observations. P-values less than 5% level of significance were considered significant. All analyses were performed using SAS 9.2 (SAS Institute Inc., Cary, NC, USA.).

## Results

### Healthcare encounters and cost analysis

**Figure 2:**
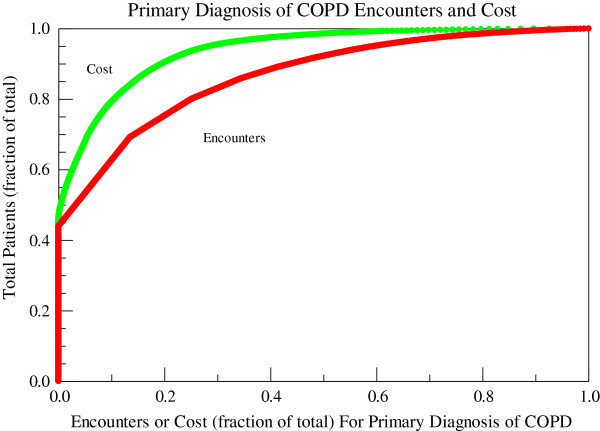
**Encounters and costs for a primary diagnosis of COPD at the cincinnati VAMC in FY08.** Costs are represented by the green line and encounters (outpatient visits, emergency department visits, and hospitalizations) by the red line. FY: fiscal year; COPD: chronic obstructive pulmonary disease.

**Figure 3:**
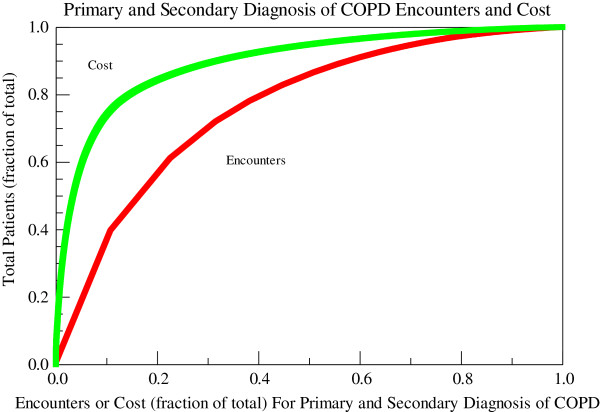
**Encounters and costs for primary and secondary diagnosis of COPD at the cincinnati VAMC in FY08.** Costs are represented by the green line and encounters (outpatient visits, emergency department visits, and hospitalizations) by the red line. FY: fiscal year; COPD: chronic obstructive pulmonary disease.

3263 unique individuals with age 67 ± 11 years (mean ± SD) were diagnosed with and received care for COPD at the Cincinnati VAMC in FY08. These individuals had 11,869 encounters with a primary or secondary diagnosis of COPD: 10,032 clinic visits (3.07 ± 0.08 visits/person), 505 emergency department (ED) visits (0.15 ± 0.01 visits/person), and 1,332 hospitalizations (0.410 ± 02 hospitalizations/person) and incurred a total COPD-related healthcare cost of $21.4 M: $2.4 M clinic visits, $0.21 ED visits, and $18.7 M hospitalizations and $0.89 M for COPD-related prescription costs. Veterans with COPD had 3.64 ± 0.09 total COPD-related encounters per person with a mean cost of $6546 ± 302/person. The median total COPD-related healthcare cost per person was $1004 (417, 4041) (median (25th and 75th percentiles)). 2877 Veterans (88.2%) had at least one clinic visit whereas only 876 (26.8%) had an admission with a primary or secondary diagnosis of COPD. Of those Veterans who had at least one clinic visit for COPD, the average number of visits was 3.49 ± 0.09 visits/person and the median was 2 visits/person (range 1–56). For those patients with at least one admission, the mean number of admissions was 1.5 ± 0.2 admissions/person and the median was 1 admission/person (range 1–11). The distributions of costs and encounters for a primary diagnosis of COPD and for primary and secondary diagnosis of COPD are shown in Figures 2 and 3. When the patients were ranked by VHA healthcare costs, the top 20% of patients accounted for 86% of the total costs and 57% of the total encounters with a primary or secondary diagnosis code of COPD and 90% of the total costs and 75% of the total encounters with a primary diagnosis code of COPD. All hospitalizations and ED visits and 69% of outpatient encounters with a primary diagnosis of COPD occurred in this top quintile.

### Demographics

The demographics and clinical characteristics of the study population are presented in Tables 1, 2, and 3.

**Table 1:** Summary of categorical demographic and clinical characteristics

**Variable (Missing n)**	**n (%)**
Gender	
Male	816 (97%)
Female	24 (3%)
Marital Status	
Single	45 (5%)
Married	400 (48%)
Divorced	272 (32%)
Separated	31 (4%)
Widowed	92 (11%)
Race (41)	
White	696 (87%)
African American	103 (13%)
Smoking (265)	
Never	36 (6%)
Past	260 (45%)
Current	279 (49%)
Comorbidities	
Coronary Artery Disease	240 (29%)
Depression	204 (24%)
Diabetes Mellitus	181 (22%)
Heart Failure	45 (5%)
Hypertension	527 (63%)
Vaccination	
Influenza	620 (74%)
Pneumococcal	722 (86%)
Medications	
Long acting beta agonist	271 (32%)
Short acting anti-muscarinic	473 (56%)
Long acting anti-muscarinic	124 (15%)
Inhaled corticosteroid	226 (40%)
Oral corticosteroid	39 (5%)
Supplemental oxygen	196 (23%)
Theophylline	38 (4%)
Statin	467 (56)
Illicit drug use (303)	91 (17%)
Nursing home resident	36 (4%)
Treated by a pulmonologist	216 (26%)
COPD for > 5 years	260 (31%)

(Percentage is the proportion of patients with identified clinical characteristics) COPD-chronic obstructive pulmonary disease.

**Table 2:** Summary of quantitative demographic and clinical characteristics

**Variable**	**N**	**Mean ± SD**	**Median (25^th^-75^th^ percentile)^α^**
Age (years)	840	67.29 ±10.85	66 (59–79)
Hospitalizations*	840	0.49 ± 1.00	0 (0–1)
Clinic Visits*	840	4.16 ± 5.43	2 (1–5)
ED Visits*	840	0.28 ± 0.79	0 (0–8)^β^
Number of Prescriptions*	700	10.55 ± 9.49	7 (4–15)
BMI (kg/m^2^)	834	27.87 ± 6.83	27 (23–32)
FEV1 (L)	689	1.85 ± 0.80	1.76 (1.21-2.37)
FVC (L)	689	3.23 ± 0.98	3.12 (2.56-3.82)
DLCO (ml/min/mmHg)	540	14.34 ± 6.05	13.5 (9.9-18.2)
EF (%)	390	54 ± 12	58 (53–63)
Maximum serum			
bicarbonate (mEq/L)	758	30.07 ± 3.68	30 (28–32)
Smoking (pack years)	259	62.77 ± 37.53	55 (36–80)

*Per patient; ^α^except where indicated; ^β^(minimum-maximum) *SD*, Standard deviation; *ED*, Emergency department; *BMI*, Body mass index; *FEV1*, Forced expiratory volume in 1 second; *FVC*, Forced vital capacity; *DLCO*, Diffusing capacity; *EF*, Ejection fraction.

**Table 3:** Comorbidities and Spirometry per Quintile

	**1st Quintile**	**2nd Quintile**	**3rd Quintile**	**4th Quintile**	**5th Quintile**
Comorbidity					
Depression	38 (12%)	47 (37%)	38 (31%)	40 (31%)	41 (32%)
Diabetes	31 (10%)	30 (24%)	34 (26%)	49 (38%)	36 (28%)
Coronary artery					
Disease	37 (12%)	58 (46%)	48 (37%)	53 (41%)	43 ( 33%)
Hypertension	95 (30%)	108 (85%)	99 (76%)	108 (83%)	116 (90%)
Heart failure	9 (3%)	8 (6%)	5 (4%)	15 (12%)	8 (6%)
COPD >5 years	81 (25%)	58 (46%)	33 (25%)	45 (35%)	43 (33%)
Spirometry					
FEV1 (L)	1.56 ± 0.70	1.93 ± 0.88	2.14 ± 0.76	1.98 ± 0.70	2.25 ± 0.78
FEV1% predicted	50.0 ± 20.2%	60.2 ± 22.7%	64.3 ± 19.1%	61.8 ± 18.2%	67.0 ± 20.5%
FVC (L)	3.04 ± 0.94	3.34 ± 1.07	3.43 ± 0.94	3.14 ± 0.91	3.58 ± 0.93
FVC% predicted	77.7 ± 22.4%	83.8 ± 23.0%	82.6 ± 19.5%	79.1 ± 17.4%	85.5 ± 18.1%
FEV1/FVC	50.9 ± 14.4%	56.9 ± 13.5%	62.1 ± 12.2%	62.3 ± 12.3%	62.0 ± 11.9%

Comorbidites are presented as n, percentage of the quintile; Spirometry is presented as mean ± standard deviation.

### COPD-related healthcare costs

**Figure 4:**
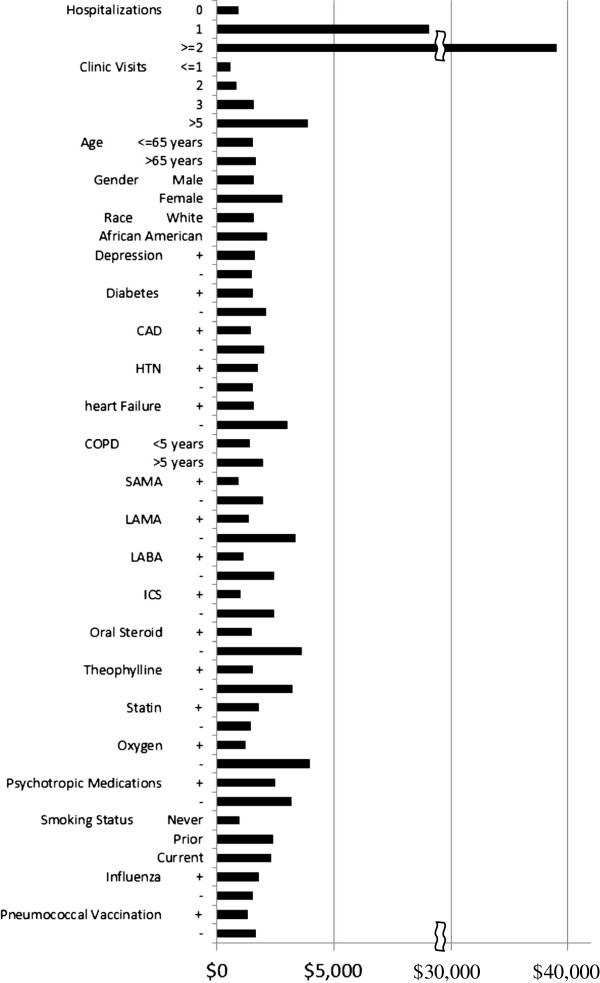
**Contribution of clinical variables to median COPD cost.** + variable present; - variable not present CAD-coronary artery disease; HTN-hypertension; COPD-chronic obstructive pulmonary disease; SAMA-short acting anti-muscarinic agonist; LAMA-long acting anti-muscarinic agonist; LABA-long acting beta agonist; ICS-inhaled corticosteroid.

The median total COPD cost according to various clinical factors is shown in Figure 4. Factors associated with COPD-related healthcare costs by univariate analysis are shown in Table 4. For every increase in the average number of admissions by one, the total average COPD cost increased by 120%; for every increase in the average number of ED visits by one, the total average COPD cost increased by 70%. Multivariable regression analysis demonstrated that 8 factors were significantly associated with total COPD-related cost (Table 5). For every increase in the average number of admissions by one, the total average COPD cost increased by 99% after adjusting for the effect of other significant cofactors. Based upon the multivariable analysis, reducing the total number of COPD admissions from the 50th percentile (2 hospitalizations/year) to the 25th percentile (1 hospitalization/year) would reduce the total COPD related healthcare cost by 6% relative to its average cost.

**Table 4:** Univariate analysis of factors associated with copd-related and total healthcare costs

	**COPD-related cost**		**Total healthcare cost**	
**Variables**	**Unadjusted regression**	**p-value**	**Unadjusted regression**	**p11-value**
	**Coefficient**		**Coefficient**	
	**[95% confidence limits]**		**[95% confidence limits]**	
Age	0.003 [−0.008, 0.013]	0.627	−0.009 [−0.017, -0.001]	0.02
Female	0.413 [−0.252, 1.077]	0.223	0.192 [−0.309, 0.692]	0.452
Married	−0.288 [−0.789, 0.213]	0.259	−0.527 [−0.904, -0.151]	0.006
Divorced	0.154 [−0.359, 0.666]	0.556	−0.222 [−0.608, 0.163]	0.258
Separated	0.010 [−0.733, 0.753]	0.979	0.004 [−0.555, 0.564]	0.987
Widowed	0.276 [−0.303, 0.856]	0.350	−0.248 [−0.684, 0.188]	0.264
Black	0.002 [−0.337, 0.341]	0.991	0.233 [−0.017, 0.483]	0.068
Current Smoker	0.750 [0.156, 1.345]	0.014	0.568 [0.14, 0.996]	0.009
Ever Smoker	0.749 [0.152, 1.346]	0.014	0.645 [0.215, 1.074]	0.003
Hospitalizations	1.196 [1.120, 1.271]	<.0001	0.747 [0.68, 0.813]	<.0001
Clinic visits	0.110 [0.091, 0.129]	<.0001	0.055 [0.04, 0.04]	<.0001
ED visits	0.695 [0.562, 0.828]	<.0001	0.356 [0.252, 0.459]	<.0001
Number of Prescriptions	0.049 [0.037, 0.060]	<.0001	0.021 [0.012, 0.03]	<.0001
BMI	−0.015 [−0.031, 0.001]	0.067	0.012 [0, 0.025]	0.048
FEV1	−0.493 [−0.639, -0.349]	<.0001	−0.019 [−0.13, 0.091]	0.73
FVC	−0.300 [−0.422, -0.178]	<.0001	−0.066 [−0.156, 0.024]	0.15
DLCO	−0.070 [−0.092, -0.049]	<.0001	−0.026 [−0.010, -0.01]	0.002
EF	−1.192 [−2.568, 0.184]	0.089	−0.545 [−1.462, 0.371]	0.243
Maximum serum bicarbonate	0.086 [0.054, 0.118]	<.0001	0.047 [0.024, 0.069]	<.0001
CAD	0.282 [0.038, 0.527]	0.024	0.27 [0.086, 0.453]	0.004
Depression	−0.102 [−0.360, 0.157]	0.440	0.281 [0.088, 0.475]	0.004
DM	0.194 [−0.075, 0.463]	0.157	0.416 [0.215, 0.617]	<.0001
Heart failure	0.422 [−0.069, 0.914]	0.092	0.541 [0.172, 0.91]	0.004
Hypertension	−0.119 [−0.348, 0.110]	0.309	0.137 [−0.035, 0.309]	0.119
Influenza vaccine	−0.083 [−0.335, 0.168]	0.516	0.037 [−0.153, 0.227]	0.704
Pneumococcal vaccine	0.147 [−0.172, 0.466]	0.365	0.058 [−0.182, 0.298]	0.633
Long acting beta agonists	0.776 [0.545, 1.007]	<.0001	0.178 [0, 0.355]	0.05
Short acting anti-muscarinic	0.756 [0.539, 0.974]	<.0001	0.131 [−0.037, 0.298]	0.126
Long acting anti-muscarinic	0.964 [0.658, 1.269]	<.0001	0.394 [0.161, 0.627]	0.001
Inhaled corticosteroids	0.815 [0.596, 1.034]	<.0001	0.196 [0.27, 0.365]	0.023
Oral corticosteroids	1.034 [0.513, 1.556]	<.0001	0.618 [0.225, 1.011]	0.002
Supplemental oxygen	1.081 [0.829, 1.333]	<.0001	0.551 [0.358, 0.745]	<.0001
Theophylline	0.619 [0.088, 1.150]	0.022	0.107 [−0.293, 0.507]	0.599
Statin	−0.128 [−0.350, 0.095]	0.262	0.133 [−0.034, 0.301]	0.118
Illicit drug use	−0.014 [−0.407, 0.380]	0.946	0.005 [−0.267, 0.276]	0.972
Nursing home resident	2.363 [1.839, 2.887]	<.0001	1.682 [1.286, 2.078]	<.0001
Treated by a pulmonologist	0.639 [0.389, 0.889]	<.0001	0.359 [0.17, 0.549]	<.001
COPD for > 5 years	0.335 [0.097, 0.574]	0.006	0.042 [−0.139, 0.222]	0.651

*ED*, Emergency department; *BMI*, Body mass index; *FEV1*, Forced expiratory volume in 1 second; *FVC*, Forced vital capacity; *DLCO*, Diffusing capacity; *EF*, Ejection fraction; *CAD*, Coronary artery disease; *DM*, Diabetes mellitis; *COPD*, Chronic obstructive pulmonary disease.

**Table 5:** Multivariable analysis of factors associated with copd-related and total healthcare costs

	**COPD-related cost**		**Total health care cost**	
**Variables**	**Adjusted regression**	**p-value**	**Adjusted regression**	**p-value**
	**coefficient**		**coefficient**	
	**[95% confidence limits]**		**[95% confidence limits]**	
Hospitalizations	0.988 [0.926, 1.050]	<0.001	0.609 [0.543, 0.674]	<0.001
Clinic visits	0.053 [0.040, 0.067]	<0.001	0.027 [0.015, 0.039]	<0.001
Treated by a pulmonologist	0.299 [0.133, 0.464]	<0.001	0.208 [0.053, 0.363]	0.009
Number of medications	0.012 [0.003, 0.021]	0.011		NS
FEV1	−0.133 [−0.234, -0.031]	0.011		NS
Short acting anti-muscarinic	0.246 [0.100, 0.393]	0.001		NS
BMI	−0.012 [−0.023, -0.001]	0.025		NS
Inhaled corticosteroid	0.168 [0.005, 0.331]	0.043		NS
DM		NS	0.291 [0.134, 0.449]	<0.001
Depression		NS	0.195 [0.040, 0.351]	0.014
Age		NS	−0.008 [−0.014, -0.002]	0.012
Nursing home resident		NS	0.506 [0.104, 0.909]	0.014
Maximum serum bicarbonate		NS	0.021 [0.003, 0.039]	0.026

*FEV1*, Forced expiratory volume in 1 second; *BMI*, Body mass index; *DM*, Diabetes mellitus; *NS*, Not significant.

### Total healthcare costs

Univariate analysis revealed 22 variables that were significantly associated with total healthcare costs (Table 4). Multivariable regression analysis revealed 8 variables that were significantly related to total healthcare costs (Table 5). Diffusing capacity (DLCO), smoking status, and ejection fraction were excluded from these analyses because of the amount of missing data.

## Discussion

Our study cohort of 3263 patients represented 9.3% of the approximately 35,000 Veterans receiving care at the Cincinnati VAMC during FY 2008. This estimated prevalence of diagnosed COPD compares well with the 8.2% prevalence rate among Veterans in the southeastern US (Veterans Integrated Service Network 16) calculated by Sharafkhaneh and colleagues [26] and the 9.40% prevalence of diagnosed COPD within the VHA nationally in 1999 estimated by Yu and coworkers [27]. Previously, we demonstrated that the prevalence of measured airflow limitation was significantly greater than the prevalence of diagnosed COPD and that two thirds of Veterans with airflow limitation did not have a diagnosis of COPD [1]. Assuming that only one third of patients with COPD are diagnosed, the prevalence of diagnosed Veterans with COPD, 9.3%, is comparable to our previously measured 43% prevalence of airflow limitation using a threshold of FEV_1_/FVC < 0.7.

The mean cost of COPD related care was $6546 with a median cost of $1004 (417, 4041) (25th and 75th percentiles) per patient annually. The Confronting COPD survey estimated the annual cost of COPD per patient per year in the US as $5646 [28]. An analysis of US Medicare beneficiaries in 2004 demonstrated that the attributable cost of COPD averaged about $6300 and that patients with COPD were more likely to use healthcare services and had excess total healthcare costs that were $20,500 greater than a matched comparison cohort [29]. In a study of Veterans in the southeastern US, Sharfakhaneh and colleagues [26] calculated the average cost of COPD management to be $4,437 per year whereas Yu and coworkers [27] estimated the average annual cost per patient with COPD within the VHA in 1999 to be $10,618. Studies in other healthcare systems have estimated the cost of care for patients with COPD to be $11,841/year among US Medicare recipients, and $11,580 /year in the Texas Medicaid healthcare system. Patients with COPD consume between 1.33 and 3.4 fold more healthcare resources compared with similar patients who do not have COPD [30]. Similarly, Maryland Medicaid recipients utilized 1.33-fold more healthcare resources and had 1.8-fold more adjusted average inpatient claims than controls [31].

Our study and these prior studies examined healthcare utilization in patients with a diagnosis of COPD. However, the vast majority of patients with COPD are undiagnosed. In a retrospective case–control study of healthcare utilization prior to the diagnosis of COPD, undiagnosed patients required 1.5-1.6-fold more hospitalizations, ED visits, and office visits than control patients and the costs increased over the 36 months preceding diagnosis, often increasing dramatically just prior to the diagnosis of COPD [32]. Similarly, in an analysis of 6,846 patients in the Lovelace Healthplan, Albuquerque, NM, Mapel and coworkers [33] showed that total costs were higher by an average of $1182 per person in the two years before COPD diagnosis and $2,489 in the year before diagnosis. Outpatient and pharmacy costs increased in the months just before the diagnosis of COPD was established [33]. Thus, since approximately 2/3 of Veterans with COPD are not diagnosed and the cost of care for these individuals is significantly increased, our study and others that only utilized patients with a diagnosis of COPD severely underestimate the true cost of COPD within the VHA.

Utilization of healthcare costs is not uniformly distributed among individuals with COPD. We showed that the top quintile of patients with COPD (653 of 3263 patients with COPD) incur the vast majority of the costs and all hospitalizations and ED visits associated with COPD (Figure 2). Similarly, Simon-Tuval and colleagues [30] showed that the top quartile of 398 patients with COPD consumed 63% of all costs. This distribution of healthcare expenditures is very similar to the general US population where the top 5% of the population accounted for 49% of all health care expenditures in 2002 [34]. Within the VHA in 1999, 35% of Veterans had 3 or more of the most prevalent chronic conditions and accounted for 73% of the total VHA healthcare budget [27].

Similar to other investigators, we found that hospitalizations were the greatest contributor to total COPD costs and accounted for 87% of total COPD related costs (Figure 4). Other studies have estimated that between 40 and 75% of the total cost of COPD management is due to hospitalizations for exacerbations [8,35-37]. Differences in cost accounting may explain some of these differences. Many of our patients had multiple hospitalizations for COPD exacerbations; one patient had 11 admissions in 12 months. Among Medicare beneficiaries in 15 states, approximately 20.5% of all admissions for COPD were followed by readmission within 30 days with a mean cost of $7,100 for the index admission and $10,900 for readmissions for which COPD was a primary or secondary diagnosis [38]. Approximately 63% of the total costs associated with management of COPD exacerbations are due to further treatment after failure of initial management [39]. Thus, reduction of readmissions for treatment failure after hospitalization for a COPD exacerbation may profoundly reduce COPD related healthcare costs.

Other factors associated with increased COPD-related healthcare costs included FEV_1_ which was inversely related to total COPD cost. Most COPD guidelines classify COPD severity based upon FEV_1_. Several studies have shown that COPD related healthcare costs increase as the severity of disease worsens [8,40-42]. Hilleman and colleagues showed that the cost of care rose as lung function declines, $1,681 for stage I, $5,037 for stage II, and $10,812 for stage III COPD [8]. We also showed that treatment by a pulmonologist was associated with higher COPD related costs. Patients with pulmonary consultations had worse lung function (FEV_1_, 49% v. 61%) and lower DLCO (37% v. 47%) than patients not cared for by a pulmonologist. Similarly, higher COPD related costs were associated with the use of more medications, especially short acting anti-muscarinics and inhaled corticosteroids. (At the time of this study, long acting anti-muscarinics were not on the Cincinnati VAMC formulary.)

In contrast with other studies, we did not find associations between COPD cost and comorbid illnesses, including coronary artery disease, heart failure, diabetes, and depression [30]. Diabetes and depression were, however, associated with greater total healthcare costs. Possible explanations for these differences include different study populations; Simon-Tuval and associates [30] studied predominantly enrollees of the Clalit Health Services, the largest health maintenance organization in Israel whereas our population was Veterans receiving healthcare through the VHA. Their population was diagnosed with COPD clinically and included a larger proportion of women.

### Limitations

Due to differences in patient populations, study design, and cost accounting, it is difficult to compare our results with other studies. Our population, being within the VHA, is predominately male, white, and had an extensive smoking history. One other study examining costs among Veterans with COPD [26] included patients with bronchiectasis and allergic alveolitis (hypersensitivity pneumonitis). Thus, differences in study population may account for some of the differences in results. Our study was a single center retrospective review and the findings will need to be extended to a larger national population. We only measured healthcare utilization within the VHA and did not capture nonVHA healthcare costs. Consequently our findings may underestimate the total COPD related healthcare costs of this population.

## Conclusions

When the patients were ranked by VHA healthcare costs, the top 20% of patients accounted for 86% of the total costs and 57% of the total encounters with a primary or secondary diagnosis code of COPD and 90% of the total costs and 75% of the total encounters with a primary diagnosis code of COPD. This top quintile generated all ED visits and hospitalizations and the majority of office visits. The total number of admissions, clinic visits, physiologic impairment, BMI, number of medications, and type of provider were strongly associated with the total cost of COPD management. These factors may be used to improve COPD management for those patients who have the greatest potential for the highest utilization of healthcare resources.

## Competing interests

The authors declare that they have no competing interests.

## Authors’ contributions

KD participated in the design of the study, performed the chart reviews and data collection, and wrote the manuscript. AD performed the statistical analysis and assisted in writing the manuscript. ZW assisted in both the statistical analysis and writing the manuscript. RP conceived the study, participated in its design and coordination, and writing the manuscript. All authors read and approved the final manuscript.

## Authors’ information

KD and RP are affiliated with the Pulmonary, Critical Care, and Sleep Divisions at the Cincinnati VAMC and the University of Cincinnati College of Medicine; AKD and ZW are affiliated with the Division of Biostatistics and Epidemiology, University of Cincinnati College of Medicine.

## Supplementary Material

Additional file 1 **Literature review of clinical studies determining factors associated with acute exacerbations of COPD (AECOPD).** The numbers refer to the number of studies citing each factor and its relationship with AECOPD. Click here for file
