# Supplementary material for: Disproportionate utilization of healthcare resources among veterans with COPD: a retrospective analysis of factors associated with COPD healthcare cost
Source: Cost Eff Resour Alloc. 2013 Jun 13;11:13. doi: 10.1186/1478-7547-11-13 (PMC3700817; doi:10.1186/1478-7547-11-13)
Supplement: Additional file 1 — Literature review of clinical studies determining factors associated with acute exacerbations of COPD (AECOPD). The numbers refer to the number of studies citing each factor and its relationship with AECOPD. [file 1478-7547-11-13-S1.docx]

Appendix 1

| **Clinical Characteristic** | **Positive**  **Association** | **No**  **Association** | **Negative**  **Association** |
| --- | --- | --- | --- |
| **Lower Pulmonary Function** | 5 | 2 |  |
| **Prior Hospital Admissions in past year** | 5 |  |  |
| **On high does systemic corticosteroids** | 3 |  |  |
| **St. George Respiratory Questionnaire** | 2 |  |  |
| **No long-term oxygen therapy or <15hr/day when indicated** | 2 |  |  |
| **On long-term oxygen therapy** | 2 |  |  |
| **Age** | 2 | 2 |  |
| **Absence pneumococcal vaccine** | 1 |  |  |
| **Absence of physical activity** | 1 |  |  |
| **Alcohol Consumption** | 2 |  |  |
| **Consumption of sedatives** | 1 |  |  |
| **Improper use of inhalers** | 1 |  |  |
| **Current smoking** | 1 | 1 | 1 |
| **Passive smoke exposure** | 1 |  |  |
| **Occupational exposure** | 1 |  |  |
| **Flu vaccine** | 1 | 1 |  |
| **Length of hospital admission** | 1 |  |  |
| **Nursing home resident** | 1 |  |  |
| **Dependent on assistance in self care** | 1 |  |  |
| **Right heart strain pattern on EKG** | 1 |  |  |
| **Coronary Artery Disease** | 1 |  |  |
| **Left ventricular failure** | 1 |  |  |
| **On high dose inhaled corticosteroids** | 1 |  |  |
| **PaCO2>50 mmHg** | 2 |  |  |
| **Actual bicarbonate > 25 mmol** | 1 |  |  |
| **Oral theophyllines** | 1 |  |  |
| **Care controlled by pulmonologist** | 1 |  |  |
| **Anticholinergics** | 1 | 1 |  |
| **Lower PO2** | 1 | 1 |  |
| **Diagnosis of COPD >5 yrs** | 1 |  |  |
| **Consumption of psychotropic drugs** | 1 |  |  |
| **Received pneumovax** | 1 |  |  |
| **Sex** | 1 | 1 |  |
| **Increased fibrinogen** | 1 |  |  |
| **DLCO** | 1 |  |  |
| **Higher BODE index** | 1 |  |  |
| **Increased mean pulmonary artery pressure** | 1 |  |  |
| **Respiratory Virus** | 1 |  |  |
| **Socioeconomic status** |  | 2 |  |
| **Psychological status i.e. depression** |  | 1 |  |
| **Marital status** |  | 1 |  |
| **Comorbidities** |  | 1 |  |
| **BMI** |  | 1 |  |
| **Poor Compliance** |  | 1 |  |
| **Poor family support** |  | 1 |  |
| **PE/DVT** |  | 1 |  |
| **Lack of Flu vaccination** |  |  | 1 |
| **Long-term utilization** |  |  | 1 |
| **Better score on physical scale of health** |  |  | 1 |
| **Walking >60 minutes per day** |  |  | 1 |
| **Primary Care Provider** |  |  | 1 |

Literature review of clinical studies determining factors associated with acute exacerbations of COPD (AECOPD). The numbers refer to the number of studies citing each factor and its relationship with AECOPD.
